# A Pandemic within the Pandemic? Physical Activity Levels Substantially Decreased in Countries Affected by COVID-19

**DOI:** 10.3390/ijerph18052235

**Published:** 2021-02-24

**Authors:** Jan Wilke, Lisa Mohr, Adam S. Tenforde, Pascal Edouard, Chiara Fossati, Marcela González-Gross, Celso Sánchez Ramírez, Fernando Laiño, Benedict Tan, Julian David Pillay, Fabio Pigozzi, David Jimenez-Pavon, Bernhard Novak, Johannes Jaunig, Mandy Zhang, Mireille van Poppel, Christoph Heidt, Steffen Willwacher, Gustavo Yuki, Daniel E. Lieberman, Lutz Vogt, Evert Verhagen, Luiz Hespanhol, Karsten Hollander

**Affiliations:** 1 Department of Sports Medicine, Goethe University Frankfurt, 60488 Frankfurt, Germany; mohr@sport.uni-frankfurt.de (L.M.); l.vogt@sport.uni-frankfurt.de (L.V.); 2 Department of Physical Medicine and Rehabilitation, Spaulding Rehabilitation Hospital, Harvard Medical School, Charlestown, MA 02129, USA; atenforde@mgh.harvard.edu (A.S.T.); karsten.hollander@medicalschool-hamburg.de (K.H.); 3 Inter-University Laboratory of Human Movement Science, University Jean Monnet, 42000 Saint Etienne, France; pascal.edouard@univ-st-etienne.fr; 4 Department of Clinical and Exercise Physiology, Sports Medicine Unity, Faculty of Medicine, University Hospital of Saint-Etienne, 42055 Saint-Etienne, France; 5 Department of Movement, Human and Health Sciences, University of Rome “Foro Italico”, 00135 Rome, Italy; chiara.fossati@uniroma4.it (C.F.); fabio.pigozzi@uniroma4.it (F.P.); 6 ImFine Research Group, Department of Health and Human Performance, Universidad Politécnica de Madrid, 28040 Madrid, Spain; marcela.gonzalez.gross@upm.es; 7 Exercise Is Medicine, 28040 Madrid, Spain; david.jimenez@uca.es; 8 Sciences of Physical Activitiy, Sports and Health School, University of Santiago of Chile (USACH), Santiago 9170020, Chile; celso.sanchez@usach.cl; 9 Fundación Instituto Superior de Ciencias de la Salud, Buenos Aires 1885, Argentina; fernandoalainio@gmail.com; 10 Changi General Hospital, Singapore 529889, Singapore; benedict.tan.c.l@singhealth.com.sg (B.T.); mandy.zhang.j@singhealth.com.sg (M.Z.); 11 Basic Medical Sciences Department, Durban University of Technology, Durban 4001, South Africa; pillayjd@dut.ac.za; 12 MOVE-IT Research Group, Department of Physical Education, Faculty of Education Sciences, University of Cádiz, 11519 Cádiz, Spain; 13 Institute of Human Movement Science, Sport and Health, University of Graz, 8010 Graz, Austria; bernhard.novak@uni-graz.at (B.N.); johannes.jaunig@uni-graz.at (J.J.); Mireille.van-poppel@uni-graz.at (M.v.P.); 14 Department of Orthopedics, University Children’s Hospital Basel, University of Basel, 4056 Basel, Switzerland; christoph.heidt@ukbb.ch; 15 School of Human Movement and Nutrition Sciences, University of Queensland, St Lucia, QLD 4067, Australia; s.willwacher@dshs-koeln.de; 16 Faculty of Mechanical and Process Engineering, Offenburg University of Applied Sciences, 77652 Offenburg, Germany; 17 Masters and Doctoral Programs in Physical Therapy, Universidade Cidade de São Paulo (UNICID), Sao Paulo 03071-000, Brazil; gustavo.yuki@hotmail.com (G.Y.); l.hespanhol@outlook.com (L.H.); 18 Department of Human Evolutionary Biology, Harvard University, Cambridge, MA 02138, USA; danlieb@fas.harvard.edu; 19 Amsterdam Collaboration on Health & Safety in Sports, Department of Public and Occupational Health, Amsterdam Movement Sciences, Amsterdam UMC, University Medical Centers-Vrije Universiteit Amsterdam, 1105 Amsterdam, The Netherlands; e.verhagen@amsterdamumc.nl; 20 Institute of Interdisciplinary Exercise Science and Sports Medicine, Medical School Hamburg, 20457 Hamburg, Germany

**Keywords:** coronavirus, health, exercise, guidelines

## Abstract

Governments have restricted public life during the COVID-19 pandemic, inter alia closing sports facilities and gyms. As regular exercise is essential for health, this study examined the effect of pandemic-related confinements on physical activity (PA) levels. A multinational survey was performed in 14 countries. Times spent in moderate-to-vigorous physical activity (MVPA) as well as in vigorous physical activity only (VPA) were assessed using the Nordic Physical Activity Questionnaire (short form). Data were obtained for leisure and occupational PA pre- and during restrictions. Compliance with PA guidelines was calculated based on the recommendations of the World Health Organization (WHO). In total, n = 13,503 respondents (39 ± 15 years, 59% females) were surveyed. Compared to pre-restrictions, overall self-reported PA declined by 41% (MVPA) and 42.2% (VPA). Reductions were higher for occupational vs. leisure time, young and old vs. middle-aged persons, previously more active vs. less active individuals, but similar between men and women. Compared to pre-pandemic, compliance with WHO guidelines decreased from 80.9% (95% CI: 80.3–81.7) to 62.5% (95% CI: 61.6–63.3). Results suggest PA levels have substantially decreased globally during the COVID-19 pandemic. Key stakeholders should consider strategies to mitigate loss in PA in order to preserve health during the pandemic.

## 1. Introduction

In March 2020, the World Health Organization (WHO) classified the spread of the novel coronavirus (SARS-CoV2) as a global pandemic. Since this declaration by the WHO, the number of laboratory-confirmed cases has grown from 125,700 (11 March 2020) to 112,205,251 (24 February 2021), while the number of affected countries has increased to 192 [1]. To control the contagion, many governments imposed substantial restrictions on public life. Initial data support the assumption that related measures (e.g., business closures, bans of social gatherings or lockdowns) and the recommendation of social distancing can effectively limit the transmission of the virus [2,3,4]. However, despite representing a crucial cornerstone to reduce the spread of viral illness, confinement strategies may have detrimental consequences for health. For instance, analyses of quarantines instituted during previous pandemics showed a variety of adverse effects such as post-traumatic stress or symptoms of depression [5].

The specific strategies used by governments to contain COVID-19 have expanded to include the closure of public parks, gyms or sport facilities and clubs. As accessibility to such areas of recreation represents an essential facilitator of physical activity (PA) [6], limitation of spaces and opportunities to move and exercise may foster sedentary behavior. Whereas inactivity has been estimated to cause up to 9% of all premature deaths [7], regular PA is well established in helping to prevent a variety of chronic non-communicable diseases such as hypertonia, metabolic syndrome, type 2 diabetes or cancer [8]. In addition to its benefits on physical health, exercise represents a valuable intervention for psychological disorders, being capable of alleviating symptoms of depression and anxiety [9,10]. In total, large-scale epidemiological studies demonstrate that 150 min/week of moderate-to-vigorous PA reduces all-cause mortality by approximately 31% [11].

Regular PA does not only play a role in general health protection. Although its direct effects on the novel coronavirus are yet to be determined, exercise, along with diet, tackles obesity, which according to initial data, seems to be a risk factor for complications in patients hospitalized for COVID-19 [12]. PA can improve immune function, e.g., via mobilizing lymphocytes and releasing cytokines such as IL-6, IL-7 and IL-15 [13]. Additionally, individuals with high activity levels are less vulnerable to infections from influenza-, rhino- or herpesviruses [13]. Particularly relevant to COVID-19, a primarily respiratory disease, research in exercise immunology has shown that PA can effectively reduce upper respiratory tract infections [14,15]. Collectively, all these data suggest that maintaining regular movement is pivotal during pandemic-related confinements.

To date, the degree to which public life restrictions related to COVID-19 affect PA levels is unknown. Early evidence based on investigations with relatively small sample sizes, however, indicate reductions of PA [16,17,18,19,20]. The present study, therefore, aimed to evaluate changes of self-reported PA in countries with SARS-CoV2 outbreaks on a multinational level.

## 2. Materials and Methods

### 2.1. Ethics and Design

Our report summarizes data from the cross-sectional ‘Activity and Health during the SARS-CoV2 Pandemic’ (ASAP) survey [21]. Ethics approval was obtained in each involved country (Australia, Austria, Argentina, Brazil, Chile, France, Germany, Italy, Netherlands, South Africa, Singapore, Switzerland, Spain, USA). All participants provided digital informed consent.

### 2.2. Sample

Participant eligibility included residents aged 18 and older from countries with (1) registered cases of SARS-CoV2 and (2) active governmental restrictions limiting movement and activity in public spaces. The recruitment strategy used social media (e.g., Facebook, Twitter, Instagram), mailing lists and health-related multipliers (e.g., national “Exercise is Medicine” chapters).

### 2.3. Instrument

The PA portion of the ASAP questionnaire assessed self-reported PA levels prior to and during restrictions of public life. To quantify PA, we applied the Nordic Physical Activity Questionnaire-short (NPAQ-short), which is reliable (test–retest reliability: rho = 0.80 to 0.82) and valid for monitoring compliance with the WHO recommendations on PA [22]. With its two questions (Table 1), the instrument retrospectively measures the times (minutes) spent performing (1) moderate-to-vigorous physical activity (MVPA) and vigorous physical activity (VPA) during leisure time. The same categories (MVPA/VPA) were used to address PA during occupational time. The NPAQ-short defines moderate activities as those increasing heartbeat and breath while vigorous activities get the affected person’s heart racing, make him/her sweat and leave him/her so short of breath that speaking becomes difficult (Table 1).

In order to achieve comparability with regard to recall periods, the participants were asked to refer (a) to the duration of confinement measures at the time of the survey for restriction PA and (b) to an identical time interval before the imposition of lockdown measures for pre-restriction PA. In addition to the NPAQ-short, we assessed changes in total PA, including light activities, by means of a five-point Likert scale (large decrease, small decrease, no decrease, small increase, large increase).

The ASAP questionnaire was generated using a group consensus process and subsequently forward- and back-translated by native speakers. To increase face validity, focus groups comprising health experts (n = 12; physicians, physiotherapists, sports scientists, movement scientists, public health advisors) and laypersons (n = 18; males and females of varying ages and educational background/socioeconomic backgrounds) within each country reviewed the survey to ensure comprehension. Following minor adjustments based on the provided feedback, the testing persons reported excellent comprehensibility and clarity.

The ASAP survey was administered via the platform SoSci Survey (SoSci Survey GmbH, Munich, Germany) between 3 April and 9 May 2020. During this timeframe, it was available for four weeks in each country.

### 2.4. Outcomes

In addition to computing changes in total leisure and occupational MVPA/VPA (min/week), compliance with the WHO’s guidelines was calculated as a dichotomous outcome (fulfilled, not fulfilled). The WHO recommends the accumulation of ≥150 min moderate activity, ≥75 min VPA or an equivalent combination of both. In addition, as the survey was performed in several countries, the Containment and Health Index (CHI [23]) was calculated for the study period. The score uses a variety of criteria to generate a score reflecting the severity of “lockdown” restrictions and measures of health protection.

### 2.5. Data Processing and Statistics

Data are reported as means including 95% confidence intervals and/or percentages, as appropriate. The normal distribution of data was verified by means of the Shapiro–Wilk test. To estimate the risk of non-response bias, wave analyses were conducted [24]. Specifically, the responses of the first 10% of the participants (early responders) in each recruitment wave were compared to those of the last 10% (late responders) by means of *t*-tests for independent samples. The rationale behind this is that early responders are assumed to be more motivated than late responders, which can be considered equivalent to non-responders. Hence, if the wave analyses do not systematically show significant findings, the absence of non-response bias is assumed. Data analyses were performed using standard statistical software packages (e.g., SPSS 22, SPSS Inc., Chicago, IL, USA and BiAs statistics, Goethe University, Frankfurt/Main, Germany). The significance level was set to α = 0.05.

## 3. Results

Participants totaled n = 13,503 (39 ± 15 years, 59% females). Fifty-four individuals reported having tested positive for SARS-CoV in the past, which is equivalent to 0.40 percent of the sample. Wave analyses yielded no indication of non-response bias (*p* > 0.05).

### 3.1. Changes in Physical Activity

**Figure 1:**
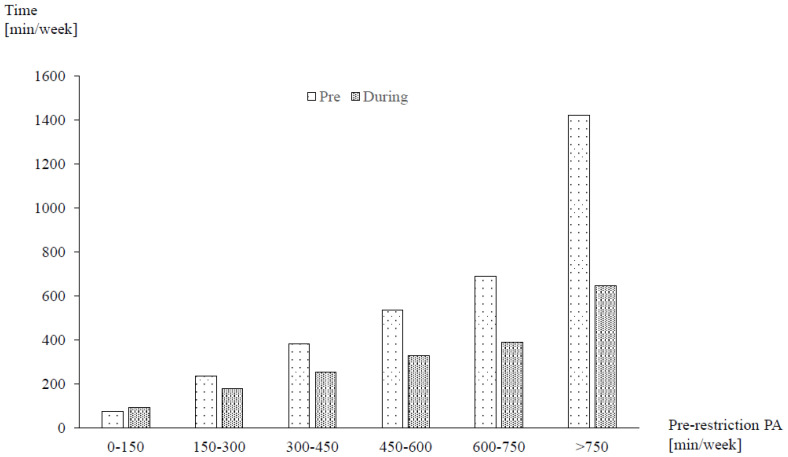
Changes in moderate-to-vigorous physical activity (MVPA) as a function of pre-restriction activity. Figure shows absolute means. PA = physical activity, min = minutes.

**Figure 2:**
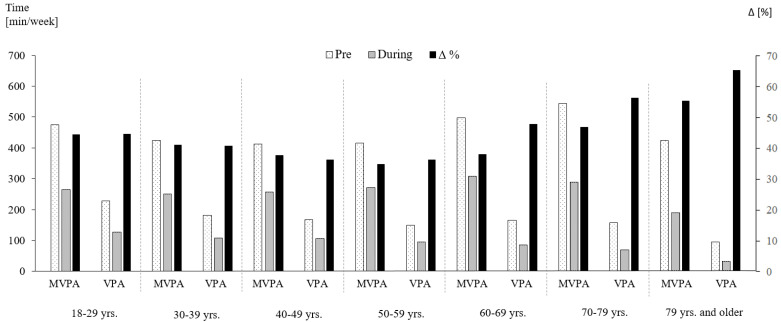
Changes in physical activity levels as a function of age. Figure shows absolute means and relative differences. min = minutes, MVPA = moderate-to-vigorous physical activity, VPA = vigorous physical activity, yrs. = years, Δ = difference.

**Figure 3:**
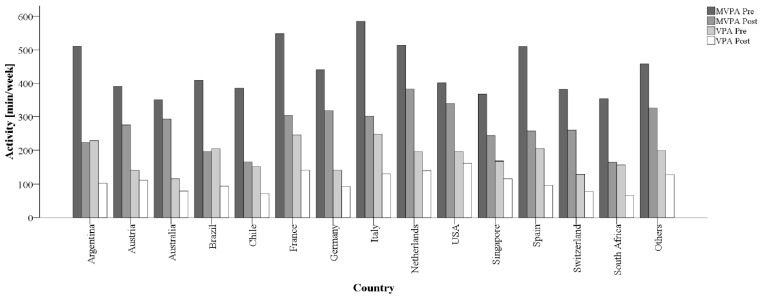
Mean changes in MVPA and VPA levels stratified by countries. min = minutes, MVPA = moderate-to-vigorous physical activity, VPA = vigorous physical activity.

**Figure 4:**
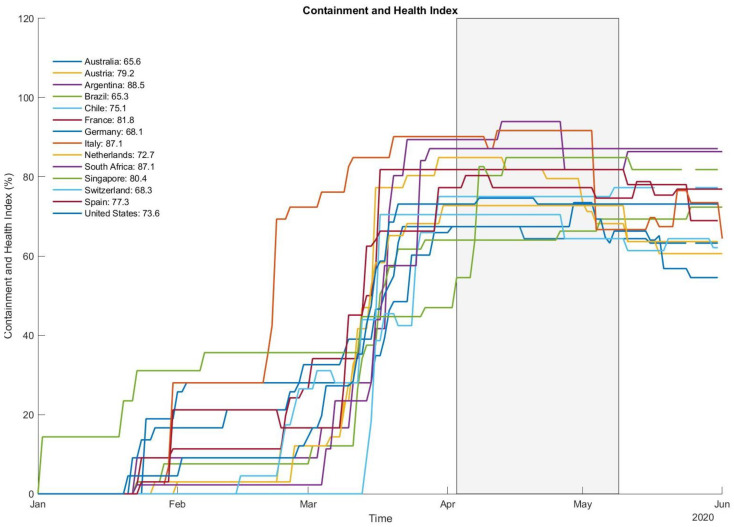
The severity of public life restrictions as measured with the Containment Health Index. Box (right) and individual country values (left) represent the period of data collection.

Mean self-reported MVPA (−41.0%) and VPA (−42.2%) both decreased to a similar degree from pre- to during restrictions (Table 2). More than two-thirds of participants (66.8%; n = 9016) were unable to maintain their previous activity levels. Declines in PA were 10% to 20% higher for occupational than for leisure time (Table 2). While there were no major differences between men and women, participants with higher pre-restriction PA had larger decreases than previously less active individuals (Figure 1). With regard to age, the highest reductions were found in the youngest and oldest participants, resembling a U-shaped distribution. Elderly individuals (70 years and older), furthermore, exhibited the highest VPA decreases (Figure 2). Significant variation was observed between countries with the highest relative reductions in Argentina, Brazil, Chile and South Africa (all > 50% in MVPA; Figure 3). Comparisons against CHI data (Figure 4) suggested a correlation between the severity of public life restrictions/health-related measures and PA reductions in most countries, although both variables seemed independent in some cases (i.e., low CHI values for Brazil, Chile and Spain despite large PA decreases). Participants with past SARS-CoV2 infection had comparable pre-restriction PA, but displayed stronger reductions in vigorous activities (−48.6% vs. −42.2%).

Regarding overall PA, including light activities, most participants (75.5%) reported decreases during restrictions, with 48.7% of them describing a large reduction. Increases from pre-restrictions were indicated in 17.7% of the participants.

### 3.2. Changes in Physical Activity Guideline Compliance

Pre-restrictions, 80.9% (95% CI: 80.3–81.7) of the sample (n = 10,938) were in compliance with the PA recommendations. Post-restrictions, the overall population fulfilling the guidelines decreased to 62.5% (95% CI: 61.6–63.3; n = 8435). More than one quarter (28.4%, n = 3104) of the participants previously meeting the required cut-offs subsequently fell short during the restrictions. In total, for the individuals who complied with the guidelines pre-restrictions, the average reported MVPA levels were reduced during the restrictions (−43.2%). In contrast, those who fell below guidelines pre-restrictions were able to increase self-reported MVPA (+56.9%), but in most cases (76.6%), this was not sufficient to achieve compliance. Participants with past SARS-CoV2 infection, compared to others, displayed massive declines in guideline compliance (88.9% to 50% vs. 80.9% to 62.5%).

Individuals losing guideline compliance during lockdowns were of similar age as those maintaining it (Table 3). However, persons newly fulfilling the guidelines were three years younger than others. With regard to sex, a slightly higher share of females gained compliance from pre- to during restrictions.

## 4. Discussion

It has been estimated that the spring 2020 public life restrictions related to the COVID-19 pandemic affected up to 3 billion persons worldwide [13]. To the best of our knowledge, this is the first multinational assessment aiming to gauge the effects of the confinements on self-reported PA. Our main finding is that the amount of habitual movement declined by 41% and 42% for MVPA and VPA, respectively. These reductions resulted in a 20% lower compliance with the 2010 WHO guidelines on PA. Taken together, our data support the myriad of calls to maintain PA during confinement [13,25,26,27] and suggest a large portion of the population may be silently suffering from a less visible impact of the pandemic on public health.

Our main result of decreases in PA aligns with available data collected in individual countries (e.g., [16,17,19,20]). The same applies to more pronounced reductions in previously active individuals (e.g., [16,17,20]). In contrast, findings are less consistent with regard to sex and age. Notwithstanding, interestingly, the reported magnitude of PA reductions here (41% to 42%) and guideline compliance declines (18.5%) was larger than reported from other surveys [16,17,18,19,20].

Sustained reductions in habitual movement can contribute to a plethora of negative health consequences. According to the World Health Survey, including responses from 237,964 individuals, physical inactivity is associated with an odds ratio of 1.32 for future states of anxiety [10]. A meta-analysis pooling the results from 49 prospective studies found persons with low activity levels to exhibit a higher risk for depression [9]. Lack of PA also results in maladaptive changes to body composition (e.g., increase in body fat), associated with decreased insulin sensitivity, reduced cardiorespiratory fitness and increased dyslipidemia [28]. These changes may occur in as little as 14 days of physical inactivity, and are reversible in young but not in older adults [29]. Considering that pandemic-related restrictions have been in place for weeks to months, the observed PA reductions, besides causing short- and long-term psychological distress, could compound the over-prevalence of non-communicable diseases. Finally, PA can be expected to have a direct beneficial impact on the COVID-19 pandemic due to its positive impact on the immune system and the risk for upper respiratory tract infections [14,15]. Wong et al. [30] analyzed influenza-associated mortality using data from Hong Kong in 1998. A low to moderate exercise frequency reduced the risk of death from influenza by 4.2% to 6.4%, while never or seldom exercising was associated with a 5.8% to 8.5% increased mortality risk. Future research should hence be geared towards investigating similar associations in COVID-19.

Our results suggest that decreases in PA particularly affect those participants who were most active prior to the pandemic. This further enhances concerns of adding to the overall proportion of the world population not meeting WHO guidelines, and, if sustained, this could contribute to rises in medical expenses resulting from disease or inactivity. An economic analysis of US data revealed that the annual healthcare costs per capita are USD 1437 and 713 higher for inactive or insufficiently active vs. active (≥150 min moderate activity per week) individuals, respectively [31]. Extrapolating this to the data of our study, this would roughly mean that only the additional healthcare costs for the 3104 persons who no longer met the PA guidelines during restrictions would translate to between USD 2,172,000 and 4,460,448 per year of continued inactivity.

Older individuals, together with young participants, had the highest reductions in PA. Declines were particularly pronounced for VPA, where adults aged 70 and older showed reductions between 56% and 67%. At matched total energy expenditure, VPA outperforms moderate PA in reducing the risk of cardiovascular disease and associated markers, such as diastolic blood pressure or glucose control [32]. In addition, data collected in middle-aged and older adults demonstrate that the engagement in VPA yields 9% to 13% lower all-cause mortality risks, when compared to identical volumes of moderate activity only [33]. In view of the older persons’ large VPA reduction during the pandemic, and given that the elderly generally have the highest risks of chronic diseases and complications following influenza infections, this target group needs special consideration [26].

The novel report on reductions in PA has broad implications for key health-related stakeholders and policymakers. When imposing pandemic-related restrictions on public life, we suggest the development of strategies to proactively counteract the anticipated inactivity. This may be achieved through public education, facilitating PA opportunities at tolerable viral transmission risk and interventions that can be rapidly implemented. Regarding the latter strategies, home-based exercise programs, offered by certified health and exercise professionals, could represent a low-cost option to maintain PA levels while being restricted to the home.

There are some methodological issues that merit consideration. The WHO has recently updated its guidelines on recommended PA levels. It is now advised to engage in a minimum of 150 to 300 (previously: 150) minutes of moderate activity, a minimum of 75 to 150 (previously: 75) minutes of vigorous activity or an adequate combination of both. Despite these new changes, our classification of individuals complying or not complying with the guidelines, which is based on the 2010 recommendations, is still valid, because the lower margin (150 min of moderate and 75 min of vigorous activity) was maintained in the new version. The absence of non-response bias and large sample size are two major strengths of the present paper, as they improve the generalizability of the findings to understanding changes in PA. Further, our sample was close to the representative pre-restriction PA levels reported: baseline compliance (81%) with WHO recommendations was only slightly higher than the pooled percentage (73%) of 358 previous surveys, including a total of 1.9 million participants [34]. However, it should also be noted that determining response rates is difficult with social media recruitment, and it is possible that persons with low internet affinity or limited access to technology did not participate in our investigation. Another issue relates to the mode of outcome assessment. Like most large-scale studies assessing PA, we used self-reported data instead of objective instruments such as accelerometers. Typically, subjective measures tend to overestimate the actual PA levels, and moderate activities are recalled less precisely than vigorous activities [35]. Although we found substantial PA decreases in both MVPA and VPA, this should be considered when interpreting our results. Finally, our analysis focused on the changes of PA and a few potential moderators, including age, sex, country of origin and baseline physical activity. However, in addition to these, an impact of other factors such as the living environment (urban vs. rural), educational level or socioeconomic status seems highly plausible. Future studies may hence consider jointly assessing these factors in conjunction with the variables presented here.

## 5. Conclusions

Self-reported PA substantially decreased following public life restrictions associated with the COVID-19 pandemic. In view of the short- and long-term consequences of inactivity, such as impaired mental and physical wellbeing, a possible higher susceptibility to viral infections and increased risk of non-communicable diseases, the implications of our findings warrant careful consideration by governmental and health-related decision-makers.

## Figures and Tables

**Table 1:** Items of the Nordic Physical Activity Questionnaire (short form) as used in the survey.

**Physical Activities in Leisure Time** We would like to know, how physically active you have been in your **free time** (including commuting from and to work). We only ask about moderate and vigorous activities—light activities do not need to be reported here. Moderate activities are those where your heartbeat increases and you breathe faster (e.g., brisk walking, cycling as a means of transport or exercise, heavy gardening, running or recreational sports). Vigorous activities as those that get your heart racing, make you sweat and leave you so short of breath that speaking becomes difficult (e.g., swimming, running, cycling at high speeds, intensive cardio training, weight-lifting or team sports such as football).	
**Physical Activities in the Job** While the previous questions addressed free time, the following two focus on **work/occupational time**. Again, we only ask about moderate and vigorous activities—light activities do not need to be reported here. Remember: Moderate activities are those where your heartbeat increases and you breathe faster (e.g., brisk walking). Vigorous activities are those that get your heart racing, make you sweat and so short of breath that you find it difficult to speak (e.g., repeated lifting of heavy weights).	
**Moderate and Vigorous Activities** On a typical week, how much time in minutes did you spend in total on both moderate and vigorous physical activities? Please sum all activities with a minimal duration of 10 min. Enter 0, if there was not at least one activity of more than 10 min. before lockdown: ___ minutes during lockdown: ___ minutes	**Vigorous Activities only** How much of that time in minutes you indicated above, did you spend in total on vigorous physical activities only? Please sum all activities with a minimum duration of 10 min. Enter 0, if there was not at least one activity of more than 10 min. before lockdown: ___ minutes during lockdown: ___ minutes

For the two NPAQ-short questions (white background), applied for free and occupational time, respectively, specific introductions (gray background) were used.

**Table 2:** Physical activity levels of the investigated sample pre- and during the restrictions.

	Leisure		Work		Total	
	MVPA (min/wk)	VPA (min/wk)	MVPA (min/wk)	VPA (min/wk)	MVPA (min/wk)	VPA (min/wk)
Pre	296.0 (290.6 to 301.5)	134.7 (131.4 to 138.0)	154.1 (148.0 to 160.2)	54.4 (51.2 to 57.5)	450.1 (440.7 to 459.6)	189.1 (183.6 to 194.5)
During	193.7 (189.6 to 197.7)	81.9 (79.5 to 84.2)	72.1 (68.4 to 75.8)	27.5 (25.5 to 29.5)	265.8 (259.7 to 271.9)	109.4 (105.7 to 113.0)
∆	−102.4 (−107.2 to −97.6)	−52.8 (−55.4 to −50.2)	−82.0 (−86.8 to −77.2)	−26.9 (−29.3 to −24.4)	−184.4 (−192.3 to −176.5)	−79.7 (−84.0 to −75.4)
% ∆	−34.6	−39.2	−53.2	−49.5	−41.0	−42.2

Table lists means and 95% confidence intervals (CI). MVPA = moderate-to-vigorous physical activity, min = minutes, VPA = vigorous physical activity, wk = week, ∆ = difference.

**Table 3:** Age and sex as a function of changes in physical activity guideline compliance between pre- and during restrictions.

Guideline Compliance				
	Pre and During	Pre but Not During	Not Pre but During	Neither Pre nor During
n	7834	3104	601	1964
Age	39 ± 15	38 ± 15	35 ± 13	38 ± 16
Sex	Females: 55.1% Males: 62.4%	Females: 22.8% Males: 23.2%	Females: 5.4% Males: 3.2%	Females: 16.8% Males: 11.3%

## Data Availability

Data can be made available upon request.
